# Transcriptional profiling of fetal hypothalamic TRH neurons

**DOI:** 10.1186/1471-2164-12-222

**Published:** 2011-05-10

**Authors:** Magdalena Guerra-Crespo, Carlos Pérez-Monter, Sarath Chandra Janga, Santiago Castillo-Ramírez, Rosa María Gutiérrez-Rios, Patricia Joseph-Bravo, Leonor Pérez-Martínez, Jean-Louis Charli

**Affiliations:** 1 Departamento de Genética y Fisiología Molecular, Instituto de Biotecnología, Universidad Nacional Autónoma de México (UNAM), Cuernavaca, Morelos, México; 2 Laboratory of Molecular Biology, Medical Research Council, Hills Road, Cambridge CB2 0QH, UK; 3 Centro de Ciencias Genómicas, Universidad Nacional Autónoma de México (UNAM), Cuernavaca, Morelos, México; 4 Departamento de Microbiología Molecular, Instituto de Biotecnología, Universidad Nacional Autónoma de México (UNAM), Cuernavaca, Morelos, México; 5 Departamento de Medicina Molecular y Bioprocesos, Instituto de Biotecnología, Universidad Nacional Autónoma de México (UNAM), Cuernavaca, Morelos, México; 6 Institute for Genomic Biology, University of Illinois at Urbana-Champaign, Urbana, 61801, USA

## Abstract

**Background:**

During murine hypothalamic development, different neuroendocrine cell phenotypes are generated in overlapping periods; this suggests that cell-type specific developmental programs operate to achieve complete maturation. A balance between programs that include cell proliferation, cell cycle withdrawal as well as epigenetic regulation of gene expression characterizes neurogenesis. Thyrotropin releasing hormone (TRH) is a peptide that regulates energy homeostasis and autonomic responses. To better understand the molecular mechanisms underlying TRH neuron development, we performed a genome wide study of its transcriptome during fetal hypothalamic development.

**Results:**

In primary cultures, TRH cells constitute 2% of the total fetal hypothalamic cell population. To purify these cells, we took advantage of the fact that the segment spanning -774 to +84 bp of the *Trh *gene regulatory region confers specific expression of the green fluorescent protein (GFP) in the TRH cells. Transfected TRH cells were purified by fluorescence activated cell sorting, various cell preparations pooled, and their transcriptome compared to that of GFP- hypothalamic cells. TRH cells undergoing the terminal phase of differentiation, expressed genes implicated in protein biosynthesis, intracellular signaling and transcriptional control. Among the transcription-associated transcripts, we identified the transcription factors Klf4, Klf10 and Atf3, which were previously uncharacterized within the hypothalamus.

**Conclusion:**

To our knowledge, this is one of the first reports identifying transcripts with a potentially important role during the development of a specific hypothalamic neuronal phenotype. This genome-scale study forms a rational foundation for identifying genes that might participate in the development and function of hypothalamic TRH neurons.

## Background

The hypothalamus mediates homeostasis by integrating various endocrine and autonomic responses. Distinct nuclei of the hypothalamus regulate sleep, circadian rhythm, energy homeostasis, sexual behaviors and thermogenesis. Despite extensive studies on the physiological and clinical aspects of hypothalamic function, the molecular mechanisms defining the identity of the neuronal subtypes within each hypothalamic nucleus during development remain poorly defined.

In the murine hypothalamus, five of the neuroendocrine phenotypes [corticotropin-releasing hormone (CRH), thyrotropin releasing hormone (TRH), somatostatin (SS), growth hormone releasing hormone (GHRH), and dopamine (DA) neurons] are generated during partially overlapping periods of time, mainly from the proliferative neuroepithelium of the third ventricle [1]. Cells that produce CRH are generated between embryonic (E) days 12 and E14, with the peak generation at E13. DA and SS neurons are generated between E11 and E17, while the GHRH and TRH neurons are generated between E11 and E15, with the peak generation at E11 and E13, respectively [2-4].

An interesting observation is that subpopulations of neuroendocrine cells coexisting in the same hypothalamic nucleus produce different neuropeptides (e.g. TRH and CRH) [5,6]. The distinct neurotransmitter phenotypes do not differ in time of generation and may appear in response to individual differentiation programs involving specific gene networks, as demonstrated for serotoninergic, noradrenergic or dopaminergic phenotypes [7].

The development of the central nervous system (CNS) is achieved through a delicate balance between cell proliferation, subsequent cell cycle withdrawal and differentiation to distinctive neuronal phenotypes. Recent observations have highlighted that both extracellular cues (growth factors, extracellular matrix, Notch-Delta signaling, N-CAMs) and intracellular signals (transcription factors) play pivotal roles in this process [8,9]. In addition, post-translational histone and/or DNA enzymatic modifications, collectively called epigenetic gene regulation, also govern the process of neurogenesis [10].

*In vivo *models provide evidence that several transcription factors belonging to the basic helix-loop-helix, homeobox and POU domain families determine the proper establishment and maturation of various neuronal phenotypes within the hypothalamic nuclei [11-13]. In spite of these observations, the inductive signals and final targets of these transcription factors are poorly characterized.

Our group has previously demonstrated that the neurotrophin brain-derived neurotrophic factor (BDNF) increases hypothalamic *Trh *mRNA expression in rat E17 primary cultures. The BDNF effect is only observed in a population of TRH neurons that express the catalytic isoform of the BDNF receptor, TrkB [14]. *In vivo *studies have also demonstrated that the expression of the TrkB receptor precedes chronologically that of TRH in the paraventricular nucleus (PVN) of the rat hypothalamus; the effect of BDNF on *Trh *mRNA expression can also be observed in primary cultures of PVN neurons [15]. BDNF likely regulates the acquisition and/or maintenance of this phenotype during development. To gain a better understanding of the genes that control differentiation of a specific phenotype in the hypothalamic neurons, we performed a genome-wide study to characterize the transcriptome of hypothalamic TRH neurons during the terminal phase of differentiation. This represented a challenge since the hypothalamic TRH cells constitute only about 2% of the total cell population. To address this issue, we took advantage of the fact that the -774/+84 bp regulatory region of the *Trh *promoter confers tissue specific expression [16] and allows expression of the green fluorescent protein (GFP) in the TRH expressing cells. Purification of the TRH cell population was performed by fluorescence activated cell sorting (FACS) as described previously [17].

In this report, we show that hypothalamic TRH neurons undergoing the terminal phase of differentiation, expressed genes implicated in protein biosynthesis, intracellular signaling, and transcriptional regulation. Among the transcripts enriched in the TRH neurons, we identified three potentially relevant transcription factors: the Krüppel-like factor 4 (Klf4), the transforming growth factor beta [TGFß] inducible early growth response factor (Klf10), also known as Tieg1, and the activating transcription factor 3 (Atf3). To our knowledge, this is the first report identifying these transcription factors during hypothalamic development. Current experiments in our group have shown that Klf4 and Klf10 regulate *Trh *gene expression. We provide a molecular toolkit via a compendium of expression data that can help unravel mechanisms of hypothalamic TRH neuron development.

## Results

### Enrichment of embryonic hypothalamic TRH neurons

**Figure 1:**
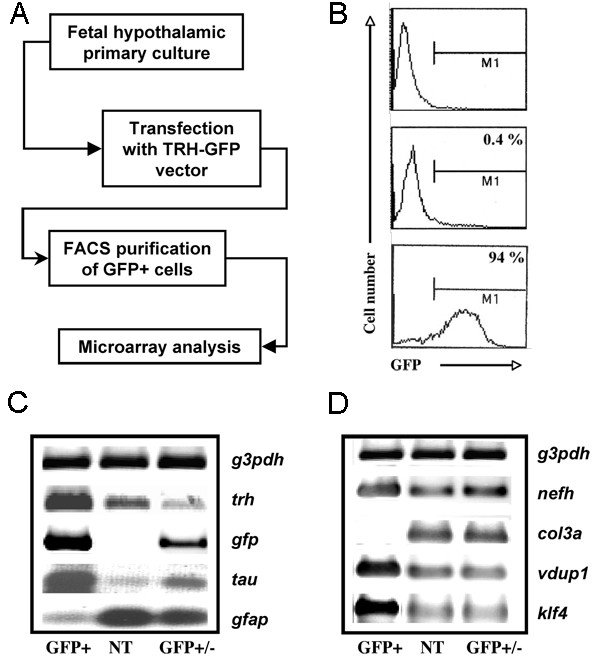
**Characterization of the TRH/GFP+ cell population in primary cultures of rat fetal hypothalamic cells**. **A**) Strategy to obtain a neuronal population enriched in TRH cells from primary cultures of fetal (E17) hypothalamic cells. Primary cultures were transfected with a vector driving GFP expression under the control of the *Trh *promoter (TRH-GFP; referred as phrTRH-GFP in materials and methods) and GFP+ cells were sorted by flow cytometry. GFP+ cells were used to generate the target cRNA to hybridize the U34A array. Non-sorted hypothalamic cells and non-transfected cells were used as control. **B**) Representative FACS plots indicating the number of GFP+ cells (M1) in a logarithmic scale for 1 × 10^4 ^events. The upper panel indicates the distribution of fluorescence in the non-transfected cell population; the middle panel indicates the distribution of fluorescence in cells transfected with phrTRH-GFP; the percentage of GFP+ cells is indicated on the top. Lower panel: after GFP+ cells were isolated by preparative FACS, they were submitted to a second pass through the FACS to determine the percentage of GFP+ cells in the enriched population; it rose to 94%. **C**) Several marker transcripts were amplified by RT-PCR. The thyrotropin releasing hormone (*trh*) and *tau *transcripts were used as neuronal markers, the glial fibrillary acidic protein (*gfap*) as glial cell marker and the green fluorescent protein (*gfp*) for transfection control. The glyceraldehyde 3-phosphate dehydrogenase (*g3pdh*) was used as a control. **D**) Analysis of transcripts identified in the GFP+ cell population according to microarray data: neurofilament heavy polypeptide (*nefh*), vitamin D3 up-regulated transcript (*vdup1*), and krüppel-like factor 4 (*Klf4*); the *g3pdh *was used as internal control. GFP+, transfected purified cells; GFP+/-, transfected non-purified cell population; NT, non-transfected cell population.

To obtain information about the transcriptome of developing TRH expressing cells, we induced GFP expression in TRH neurons using transfected primary hypothalamic cultures derived from rat embryos of 17 days of gestation. This stage corresponds to the terminal phase of differentiation of the TRH phenotype in the hypothalamus [18]. TRH neurons were enriched by FACS. The transcriptome of the TRH neurons and hypothalamic cells was determined by DNA microarray technology (Figure 1A).

We have previously reported the conditions to efficiently transfect TRH neurons in serum-supplemented cultures; control experiments suggested that most GFP+ cells were TRH neurons [17]. Taking advantage of these conditions, we transfected E17 hypothalamic cultures with a GFP expression vector under the control of the minimal *Trh *promoter region (phrTRH-GFP) and determined the transfection efficiency by FACS. After 48 h of transfection, 0.4% of cells were GFP+ (Figure 1B, middle panel). Preparative cell sorting followed by FACS analysis of the GFP+ cell population demonstrated a strong enrichment with approximately 94% of cells being GFP+ (Figure 1B, lower panel). In general, cell viability was higher than 90% in all conditions examined as determined by propidium iodide (PI) staining (data not shown).

To corroborate the neuronal identity of the sorted GFP+ cell population, the expression of *Trh *together with cell-type specific markers was examined by RT-PCR assays. GFP+ cells were separated from the GFP- cells by FACS 48 h after transfection. As a control, a mixed cell population consisting of GFP+ and GFP- cells was obtained from sorted transfected cultures without selection (GFP+/-), whereas non-transfected cells (NT) (at 3 days *in vitro*) were used to establish the basal levels of mRNA expression. An increase in *Trh *mRNA levels was observed in the GFP+ cells compared with NT cells; this was also evident with respect to GFP+/- cells (Figure 1C). The increased *Trh *expression in the isolated GFP+ cells correlated with an increase in the expression of the neuronal marker *tau*, whereas the expression of the glial cell marker *gfap *diminished (Figure 1C). As expected, *Gfp *mRNA was present only in the GFP+ and GFP+/- samples (Figure 1C). These data, together with our previous report [17], indicate that the sorting conditions permitted the enrichment of TRH neurons from a mixed primary cell culture using GFP expression as a marker of *Trh *proximal promoter activity.

### DNA microarray analysis of TRH neurons and hypothalamic cells

Once we corroborated that TRH cells were enriched in the GFP+ cell population, total RNA from GFP+ cells was isolated from a pool of six independent experiments. A pool from three other independent experiments was used to isolate total RNA from GFP+/- and NT cells. These RNA preparations were used to synthesize biotinylated cRNA targets and to screen U34A DNA microarrays.

In the array-screening experiment, two separate aliquots of GFP+ and GFP+/-RNAs, and a single one for NT RNA were used. Target generated from each aliquot was hybridized to an array, generating single (NT) and replicate (GFP+ and GFP+/-) datasets. Within the rat U34A oligonucleotide microarray, we distinguished genes based on whether or not they had a well-characterized gene name in the GenBank database. 7699 probe sets were known genes and 1130 probe sets did not have an assigned gene symbol (expressed sequence tags, ESTs); the total number of transcripts analyzed was 8829 (see http://www.ncbi.nlm.nih.gov/geo/ array).

Analysis of the signal intensity with the Microarray Suite (MAS) 5.0 software (Affymetrix) provides a statistical mean to determine the presence or absence of a gene in a sample. This test is based on the comparison of the hybridization efficiency of the target to its complementary sequence with the cross-hybridization of the target to a mismatch sequence identical to the complementary sequence except for one base. Each gene is represented by 16 probe pairs, with one of the members of each pair containing one mismatch, selected from distinct regions of the gene. The signal values from these probes were used to determine the presence of a gene in the target and a *P *value calculated from these data. A *P *value of less than 0.05 was used as a cutoff to consider that a transcript was present and a *P *value above this threshold indicated that it was absent. Transcripts were considered significantly present or absent based on the following criteria: a) the normalized value from mean differences was higher than the microarray background and, b) the fold change between match and mismatch signals was higher than 2.

On the basis of this analysis, the percentage of transcripts present in the GFP+ and GFP+/- populations was very similar. In the GFP+ cell population, 41% (2929 annotated transcripts and 583 ESTs) of the genes represented on the microarray were present, whereas 59% (4664 annotated transcripts and 398 ESTs) were absent (Additional file 1, Table S1). In the GFP+/- cell population, 41% of the genes (2941 annotated transcripts and 639 ESTs) were present and 59% (4756 annotated transcripts and 434 ESTs) were absent. In the NT cell population, 42% (3014 annotated transcripts and 571 ESTs) of the genes were present and 58% (4583 annotated transcripts and 410 ESTs) were absent.

### Enriched transcripts in the TRH neurons

To identify the set of enriched transcripts in the GFP+ population, we selected genes with a significant level of differential expression. This involved first grouping the two replicates of the control (i.e. GFP+/-) as well as that of the sorted sample's (i.e. GFP+) expression data. Signal intensities of the two replicates of control and sorted datasets were averaged to represent the expression level of a transcript in the respective control and sorted populations. These averaged intensities were used to calculate the fold enrichment in expression in sorted sample over the control for each transcript. A threshold of more than 2 fold increase or decrease in expression was considered significant to identify transcripts which are enriched in one sample but underrepresented in the other (Table 1).

**Table 1:** Specific transcripts enriched in the TRH+/GFP+ cell population in a log2-based analysis (*P *value < 0.05)

Entrez Gene ID	Gene Name	Gene symbol	log 2
	*Cytoskeleton*		
24587	Neurofilament, heavy polypeptide	Nefh	7.04
314856	Transcribed sequence similar to protein Mdm2	Mdm2	3.87
	p53 binding protein homolog		
	***Cytoplasmic***		
288656	Transcribed sequence with similarity to ADP-	Arl6ip4	1.03
	ribosylation-like factor 6 interacting protein 4		
	***Splicing events***		
114121	Cyclin L	Ccnl1	1.45
	***Cell signalling***		
24373	Follistatin	Fst	1.37
171347	Methionine adenosyltransferase II, alpha	Mat2a	1.12
304268	RAS-like family 11 member a	Rasl11a	1.17
17872	Transcribed sequence with similarity to myeloid	Myd116	1.44
	differentiation primary response gene 116		
54305	Somatostatin receptor subtype 2	Sstr2	2.8
	***Transcription***		
114505	Kruppel-like factor 4 (gut)	Klf4	2.6
25389	Activating transcription factor 3	Atf3	2.05
81813	TGFB inducible early growth response	Klf10	1.78
117514	Upregulated by 1,25-dihydroxyvitamin D-3	Txnip	1.32
314322	Rat c-fos mRNA	Fos	2.99
	***Cell cycle***		
361915	Hypothetical protein LOC361915	ND	1.04
	***Metabollic***		
154516	P glucuronosyltransferase 1 family, polypeptide A7C	Ugt1a7c	1.05
25617	Glucose-regulated protein GRP78	Hspa5	1.10
	***Neurotransmission***		
25302	Nicotinic receptor alpha 7 subunit	Chrna7	1.19
641625	Rat genes for vasopressin, oxytocin and a long interspersed repeated	ND	1.45
ND	Transcribed sequences similar to Cacnb2 and the Nsun6 gene for NOL1/NOP2/Sun domain family 6	ND	1.08
24766	Sodium channel, voltage-gated, type II, alpha 1 polypeptide	Scn2a1	2.06
	***Unknown***		
AI639457	Not similitude to any gene found	ND	2.89
U31866	Not similitude to any gene found	ND	1.59
AI639457	Not similitude to any gene found	ND	1.59
AI639215	Not similitude to any gene found	ND	1.01
M13100	Not similitude to any gene found		1.02
363278	Transcribed sequence simmilar to ATG16 autophagy related 16-like 1 (S. cerevisiae)	Atg16l1	1.08
M13100	Unknown protein	ND	1.18
M13101	Unknown protein	ND	1.29
M13100	Not similitude to any gene found	ND	1.16

ND, non determined

This analysis revealed that 30 transcripts were enriched in the GFP+ cells (Table 1). As expected, of the 30 transcripts enriched, we identified some transcripts previously associated with a neuronal phenotype, like the neurofilament heavy chain polypeptide (*Nefh*) [19], a voltage-dependent calcium channel [20], and a nicotinic alpha-receptor subunit [21]. Three of the enriched transcripts corresponded to novel transcripts within the developing hypothalamus, the Krüppel-like 4 transcription factor (*Klf4*), the TGFβ-inducible early growth response transcription factor (*Klf10*), also known as *Tieg1*, and the activator of transcription factor 3 (*Atf3*). In addition, a transcript up-regulated by vitamin D3 (*Vdup1*) was enriched in the TRH/GFP+ cell population, suggesting a potential physiological role of this vitamin within the hypothalamic TRH neurons, in agreement with previously reported data [22].

On the other hand, we found that some of the transcripts diminished in TRH/GFP+ cells (i.e. were probably not expressed in TRH cells) were associated with the glial cell phenotype. Among them are the collagen type I and type III (C*ol3a*) [23,24], and the follistatin-like gene, highly expressed in astroglial cells [25]. We also identified transcripts associated with cell cycle regulation, like annexin I, which negatively regulates cyclin D1 gene expression [26] (Additional file 1, Table S2).

**Figure 2:**
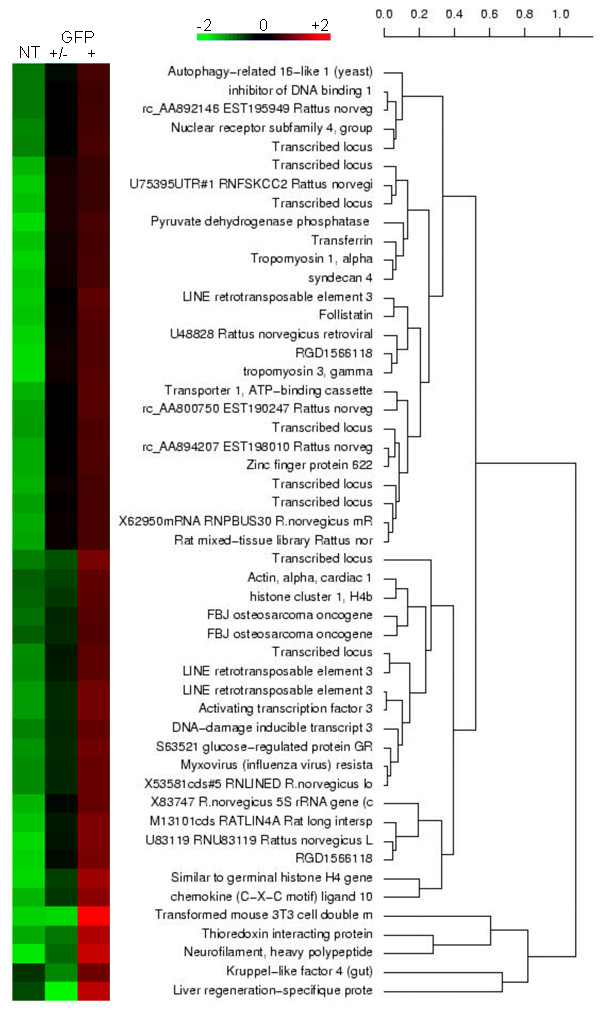
**Hierarchical clustering of differently expressed transcripts in hypothalamic cells**. The color strap indicates degrees of up- (red) and down- (green) regulation of gene expression. The dendrogram at the right shows the grouping of genes according to their similarity in expression profiles. A value of zero indicates that the genes have the same regulation profile. The transfected purified (GFP+) cell population was compared to the transfected non-purified (GFP+/-) or non-transfected (NT) cell populations in a log_2_-based threshold. The data correspond to transcripts enriched in the GFP+ cells only. The gene hierarchical cluster was performed using the multiple group analysis and the CLARA algorithm as described [54].

We then decreased the microarray threshold to 1.5 fold change to determine if any missing classes of genes can be identified in the different cell populations. We used a heat map presentation and the gene expression profile to establish a hierarchical map based on the similarity of the gene expression values. The first scale, which is associated with a coloured strap, refers to genes with up-regulated (red) or down-regulated (green) expression levels in each cell population (Figure 2). The second scale represents the degree of regulation similarity among the genes. A value of zero indicates that the transcripts have the same regulation profile. Figure 2 shows part of the transcripts identified in each cell population. This analysis confirmed the enrichment of various transcripts (*Klf4*, *Atf3*, *Nefh*) in the GFP+ cells.

To validate the microarray data, we performed RT-PCR analyses for some of the transcripts presumably enriched in the GFP+ cell population. The levels of mRNAs for *Nefh*, *Vdup1*, and *Klf4 *were increased in the purified population when compared to the NT or GFP+/- cell populations (Figure 1D). On the contrary, the glial phenotype associated transcripts, *Gafp *and *Col3a*, were absent in the GFP+ cell population (Figure 1C and 1D). These results confirmed our microarray data.

### Transcripts unique to the TRH neurons

To further breakdown the microarray data, a second method of analysis of the original signal intensities derived from the MAS software analysis was performed using a stringent *P*-value. This approach allowed us to identify transcripts present in the different cell populations with a high degree of certainty. Using a *P *value of < 0.001, we identified a total of 1864 and 1701 transcripts whose presence in the two GFP+/-replicates was significant. Similarly, we identified 1776 and 1714 transcripts in the replicate samples for the GFP+ cell population. In the NT cell population, we identified 1925 transcripts.

**Figure 3:**
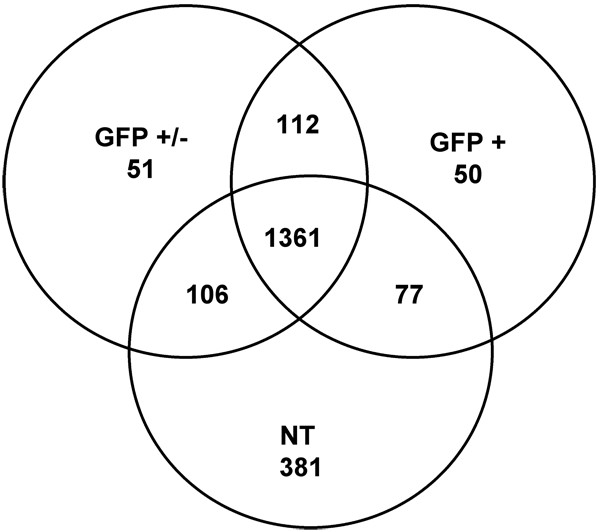
**Venn diagram showing the number of transcripts identified present in each cell population**. The circles represent a specific cell population with the numbers representing the genes present in each sample (*P *< 0.001). Numbers in the outer portion of each circle are specific to that sample. The number in the innermost portion of the diagram represents transcripts expressed in the three populations (1361). Likewise, the numbers in the portions of the diagram shared by two circles represent genes expressed in those samples but not in the exclusive sample.

In order to identify the transcripts that were present in both replicates and to reduce the false positive rate in the detection of expressed transcripts, we defined the representative set of each sample as that containing transcripts significantly expressed in both replicates according to the *P *< 0.001 threshold. This resulted in 1600 [of which 288 were ESTs (Additional file 1, Table S4)] transcripts as representative of the GFP+ cell population (Figure 3 and Additional file 1, Table S3) and 1630 transcripts for the GFP+/- cell population. As shown in Figure 3, the overlap between the three cell populations indicates that 1361 transcripts were common to the three populations, whereas 112 transcripts were common to the GFP+ and GFP+/- cell populations but not expressed in the NT cell population. This comparison also shows that 51 transcripts were unique to the GFP+/- cell population, while 50 transcripts were unique to the GFP+ cell population at these thresholds. It should be noted that in this context unique transcripts refer to those transcripts that are uniquely detected in one or more populations shown in the Venn diagram, as they are likely to be expressed in undetectable levels at these thresholds in other compared populations. According to their GenBank annotations, several of these 50 transcripts (unique to GFP+ cells) are related to neuronal phenotype, e.g. synaptojanin 1 (*Synj1*); to translation machinery, e.g. eukaryotic translation initiation factor 3 subunit 9 (*Eif3s9*), ribosomal protein L27 (*Rpl27*); to basal metabolic machinery, e.g. acyl-CoA synthetase long-chain family member 5 (*Acsl5*), solute carrier family 37 member 4 (*Slc37a4*); to cell signaling, e.g. the serine-threonine kinase 38 (*Stk38*); in addition, transcripts encoding proteins with RNA-processing properties were also observed, i.e. the nuclear transcription factor Y gamma (*Nfyc*), the splicing factor arginine/serine-rich 10 (*Sfrs10*) and the Y box protein 1 (*Ybx1*). Transcripts related to CNS development were also identified, i.e. neurofilament heavy chain polypeptide (*Nefh*), and the nuclear factor I/B (*Nifb*). A transcript with chromatin remodeling properties, the transformation/transcription domain-associated protein (*Trapp*) was also identified (Table 2). These transcripts may play a critical role in the fetal development of hypothalamic TRH neurons.

**Table 2:** Unique transcripts identified in the TRH+/GFP+ cell population using a rigorous threshold of *P *< 0

Gene ID	Gene Name	Gene symbol
S49760_g_at	140866	ND
H31982_at	ND	ND
AA849648_at	ND	ND
X83747	ND	ND
AA800750	ND	ND
AA891631	ND	ND
AI104567	Actin alpha cardiac 1	Actc1
AA892318	ADP-ribosylation factor-like 6 interacting protein 4	Arl6ip4
AA892821	Aldo-keto reductase family 7, member A2 (aflatoxin aldehyde reductase)	Akr7a2
AI639209	Ariadne homolog 2 (Drosophila) (predicted)	Arih2_predicted
AI103671	ATPase, Ca++ transporting, plasma membrane 1	Atp2b1
M60921	B-cell translocation gene 2, anti-proliferative	Btg2
AA892271	BCL2-like 13 (apoptosis facilitator) (predicted)	Bcl2l13_predicted
AI639371	Calponin 3, acidic	Cnn3
U66471	Cell growth regulator with ring finger domain 1	Cgrrf1
L23148	chr1:175179191-175179595 (+)	Y box protein 1
M13101	C-reactive protein, pentraxin-related	Crp
AI180350	CTD-binding SR-like protein rA9	LOC245925
X62160	Dynactin 1	Dctn1
AA87520	Eukaryotic translation initiation factor 3, subunit 9 (eta)	Eif3s9
AA858520	Follistatin	Fst
AI639457	GTP cyclohydrolase 1	Gch
AA799888	Mitochondrial ribosomal protein L40	Mrpl40
X62950	MRNA (pBUS30) with repetitive elements	---
AA875126	Myosin IG	Myo1g
AA818677	Neurofilament, heavy polypeptide	Nefh
AA859994	Nuclear factor I/B	Nfib
U17254	Nuclear receptor subfamily 4, group A, member 1	Nr4a1
AA875121	Nuclear transcription factor-Y gamma	Nfyc
AF040954	Protein phosphatase 1, regulatory subunit 10	Ppp1r10
AI070277	Proteolipid protein	Plp
AA875620	RGD1566118 (predicted)	---
AI176589	Ribosomal protein L27	LOC680960 Rpl27
M31018	RT1 class Ib, locus Aw2	RT1-Aw2
M10094	RT1 class Ib, locus Aw2	RT1-Aw2
AI639167	Sequestosome 1	Sqstm1
X05472	Serine incorporator 1///hypothetical protein LOC498354	LOC498354 Serinc1
AA800712	Serine/threonine kinase 38	Stk38
AF080468	Solute carrier family 37 (glycerol-6-phosphate transporter), member 4	Slc37a4
AI639294	Sparc/osteonectin, cwcv and kazal-like domains proteoglycan 2 (predicted)	Spock2_predicted
AA955859	Splicing factor, arginine/serine-rich 10 (transformer 2 homolog, Drosophila)	Sfrs10
U45479	Synaptojanin 1	Synj1
AI014169	Thioredoxin interacting protein	Txnip
AA893320	Transcribed locus (Mus musculus ubiquitin specific peptidase 7 (Usp7). Mus musculus 10 days neonate cerebellum cDNA	Usp7
AA800693	Transcribed locus, strongly similar to XP_579966.1 hypothetical protein XP_579966 [Rattus norvegicus] (Mus musculus pyridoxal (pyridoxine, vitamin B6) kinase)	---
AA800513	Transformation/transcription domain-associated protein (predicted)	Trrap_predicted
AI639488	Transformed mouse 3T3 cell double minute 2 homolog (mouse) (predicted)	Mdm2_predicted
M92074	Troponin I type 3 (cardiac)	Tnni3
AI639320	V-raf murine sarcoma 3611 viral oncogene homolog	Araf
AA894321	WD repeat and FYVE domain containing 1	Wdfy1

ND, non determined

## Discussion

Events occurring during development are tightly coupled to gene expression regulation. Some specific genes have been related to the establishment of neuronal phenotypes within the hypothalamic area. Particularly, the transcription factors Otp, Sim1, Sim2, as well as Brn2 have important roles in hypothalamic architecture (reviewed in [27,28]). The absence of either of these transcription factors during embryonic development leads to anatomical and molecular hypothalamic impairment and consequently, to the complete lack of expression of specific hypothalamic peptides [11-13]. However, the signalling pathways regulating the activity of these transcription factors and their target genes have not been established. To obtain some insight into the molecular mechanisms regulating *Trh *expression and/or TRH neuron growth during development, we determined elements of the gene expression profile of fetal hypothalamic cells enriched in TRH neurons using the DNA microarray technology. Our approach does not necessarily identify genes relevant for birth or migration, but should capture genes important for late developmental events involving TRH neuron specification and function. Here, we report that FACS enriched TRH neurons, previously cultured for 3 DIV, can be successfully used to characterize elements of their transcriptome. The database generated from this analysis allowed us to identify some transcripts, including several transcription factors, as novel candidates to regulate hypothalamic *Trh *gene expression or TRH neuron growth during the terminal phase of development. Among the transcripts enriched in the GFP+ cells, we identified three transcription factors whose expression has not been previously reported within the hypothalamus *in vivo*. These transcripts include the zinc finger domain-containing transcription factor *Klf4 *[29-32], the TGFβ-inducible early growth response transcription factor (*Klf10*) [33,34], and the activating transcription factor 3 (*Atf3*) [35]; these are important regulators of cell differentiation and proliferation in different systems. Recently, these transcription factors have been identified as NGF-responsive immediate early genes during PC12 cell differentiation [36].

Experiments performed in our group have corroborated the relevance of Klf4 for *Trh *gene expression. *Klf4 *mRNA is expressed in the embryonic rat hypothalamus, coincident with the establishment of the TRH phenotype; in the neonatal rat hypothalamus, *Klf4 *is expressed in the PVN, the source of hypophysiotropic TRH. Klf4 binds to the *Trh *promoter either *in vitro *or *in vivo *during fetal hypothalamic development. In addition, Klf4 regulates hypothalamic *Trh *promoter activity both *in vitro *and *in vivo *during development. Accordingly, *Trh *expression is down-regulated at E15 in the hypothalamus of *Klf4 *deficient mice, resulting in diminished bioactive peptide level. These data demonstrate that Klf4 is a key molecule within the differentiation program of the hypothalamic TRH phenotype [37].

TRH/GFP+ cells are enriched with another member of the Krüppel-like family of transcription factors, Klf10. Experimental evidences show that Klf10 is a positive regulator of *Trh *promoter activity in primary cultures of fetal hypothalamic cells and that its expression coincides with the establishment of the hypothalamic TRH neurons *in vivo *[38]. Klf10 binds to the *Trh *promoter *in vivo *during hypothalamus development (Martínez-Armenta M. et al. unpublished).

Previous reports have demonstrated that *Klf4*, *Klf10 *and *Atf3 *expression is regulated by TGFβ [39-41]. Since TGFβ isoforms and their receptors are expressed in the hypothalamus [42], we have analyzed the effect of this cytokine in primary hypothalamic cultures. TGFβ-2 increased *Trh *mRNA expression by regulating *Trh *promoter activity [38]. Furthermore, TGFβ-2 induced *Klf10 *mRNA in primary hypothalamic cultures similarly to *Trh *mRNA. Finally, Klf10 was bound to the rat *Trh *promoter in response to TGFβ *in vitro *(Martínez-Armenta M. et al. unpublished). Therefore, another of the transcription factors identified in the microarray screening appears critical for *Trh *expression.

In this study, we used independent preparations of GFP+ cells that were pooled to identify transcripts enriched in TRH neurons. Using independent preparations of GFP+ cells not only decreases the false positives that are possibly identified if only one preparation is used but also enriches the relative concentration of the TRH neuron specific transcripts compared to the background sample. Experimental designs using pooling samples are often performed to reduce the effects of biological variation, making substantive differences easier to find. Consequently, pooling samples has been used in many genome-wide-based studies to provide an accurate and reliable estimate of specific gene expression patterns, DNA methylation profiling, identifying biomarkers, etc. in a specific cell population [43]. Whether all the transcripts identified in this study are truly enriched in TRH hypothalamic neurons *in vivo*, and whether they are expressed into functional proteins, is not yet known, except for the evidence mentioned above for two transcripts. Under our experimental conditions, TRH neurons passed through a stage of differentiation *in vitro*; thus, the procedure used in the present study might have altered some aspects of the expression profile of the neurons, leading to some false positives. However, the results obtained with Klf4 and Klf10 (see previous paragraphs) suggest that our data should be a useful guide to study TRH neuron differentiation. However, since TRH neurons are localized in various hypothalamic nuclei [44], each identified transcript may be either enriched in a specific TRH neuron type, or in multiple types.

Previous studies have indicated that each neuronal phenotype possesses a specific differentiation program, in which in addition to transcription factors [7], epigenetic modifications and non-coding RNA expression play pivotal roles. The analysis of the transcriptome of hypothalamic TRH neurons allowed us to derive experimental data that indicate that Klf4 and Klf10 are important regulators of *Trh *gene expression during the hypothalamus development. Co-activators, such as the histone acetyltransferases (p300 and CBP), or co-repressors, such as histone deacetylases (mSin3a), can regulate *Klf4 *and *Klf10 *transcriptional activity [45]. Therefore, we propose that during hypothalamic development *Trh *gene expression is regulated by extracellular signals (i.e. TGFβ, BDNF) that modulate the accessibility of specific transcription factors (e.g. Klf4 and Klf10) to *Trh *gene promoter by local histone modifications. To gain further insight into the molecular mechanism regulating hypothalamic neuronal phenotype differentiation, it will be critical to determine the impact of specific epigenetic modifications during hypothalamus development.

## Conclusions

Although the functional importance of the hypothalamus has been demonstrated throughout vertebrates, the molecular mechanisms controlling neurogenesis in this forebrain structure are poorly understood. The hypothalamic TRH peptide has multiple hormonal and autonomous functions. Previous studies have evidenced that pituitary response to TRH is blunted in a number of psychiatric conditions, including schizophrenia, bipolar disorders, alcoholism and depression [46]. Whether specific abnormalities during the differentiation of hypothalamic TRH neurons are associated with such disorders remains unknown. Therefore, knowledge of transcriptional regulation during the course of TRH neuron differentiation might contribute to a better understanding of the molecular mechanisms underlying TRH mediated homeostasis in the adult organism. For this purpose, we performed a genome wide study of hypothalamic TRH neurons during late fetal development. We report novel transcripts within the hypothalamus that may be part of the differentiation program of the TRH neuronal phenotype. These included the transcription factors Klf4, Klf10 and Atf3. Although the role of transcription factors during neuronal differentiation is well accepted, we are only at the brink of understanding how epigenetic mechanisms influence transcriptional activity and the accessibility of transcription factors to bind to cis-elements. The identification of transcripts enriched in fetal hypothalamic TRH neurons will guide further studies on the differentiation of this phenotype.

## Methods

### Animals

Wistar rats raised at our animal facility, maintained in standard environmental conditions (lights on between 0700-1900 h, temperature 21 ± 2°C) with rat chow and tap water ad libitum. Animal care and protocols followed the guidelines for the use of animals in neuroscience research of the Society for Neuroscience, USA, and were approved by the Animal Care and Ethics Committee of the Instituto de Biotecnología, UNAM.

### Cell culture and transfection

Hypothalamic primary cultures were prepared from E17 rat embryos as previously described [17]. Briefly, pregnant Wistar rats were anesthetized with pentobarbital (33 mg/kg b.w.) and the embryos removed individually. The hypothalamus was then excised through an imaginary line between both eye and ear superior edge; the dissected area was limited by the optic chiasm and lateral sulcus including mammillary bodies to a 2-3 mm depth avoiding thalamic area. Hypothalami were dissociated with trypsin (Sigma) and viability monitored by trypan blue exclusion (95%). Cells (5 × 10^6^) were plated onto poly-D-lysine (Sigma; 10 *μ*g/ml) pre-coated 60 mm Petri dishes in DMEM supplemented with 10% fetal bovine serum (FBS, GIBCO), 0.25% glucose (Sigma), 2 mM glutamine (Sigma), 3.3 mg/ml insulin (Sigma), 1% antibiotic-antimycotic (GIBCO) and 1% vitamin solution (GIBCO). Cultures were maintained in a REVCO incubator at 37°C in humidified air/7% CO_2_.

Twenty-four hours after seeding, cells were transfected essentially as described [17]. In general, 8 mg of branched polyethylenimine (PEI) (600-1000 KDa; Fluka) solution was diluted in 10 ml of water, pH adjusted to 6.9 with 0.2 N HCl and the solution filtered (Millipore, 0.22 *μ*m). PEI (30 *μ*l) and plasmid DNA (10 *μ*g) were separately diluted to adjust NaCl to 150 mM in a final volume of 50 *μ*l, vortexed and incubated for 10 min at room temperature; subsequently, the polymer solution was added to the DNA, vortexed-mixed, incubated for 10 min at room temperature followed by the addition of 900 *μ*l of serum-free DMEM. The supplemented DMEM was removed from the culture dishes and the transfection mixture was added. After 3 hours incubation, transfection mix was removed and fresh supplemented DMEM was added. Forty-eight hours after transfection, cells were trypsinized (0.25% trypsin-EDTA) and subjected to FACS.

### Plasmid construct

The minimal *Trh *promoter (-776/+84 bp) conferring tissue-specific expression [16] was excised with EcoR1 and BamH1/EcoRV digestion from the pNASS-rTRH-Luc expression vector. The *Trh *promoter fragment sticky ends were filled with Klenow DNA polymerase and subsequently sub-cloned into the SacI/BamH1 sites present on the pACT2 vector. Finally, the *Trh *promoter fragment was cloned into the SacI/BamHI sites in the phrGFP promoterless expression vector (Stratagene) and sequenced using primers corresponding to the *Trh *promoter 3' region (5'-ATG CAT AGA TCT TCT AGA TA-3') and to the phrGFP 5'region (3'-TGC AGG CCG GTG TTC TTC AGG A-5'); the resulting plasmid was named phrTRH-GFP.

### Fluorescence-activated cell sorting

For preparative cell sorting, 5 × 10^6 ^hypothalamic cells plated on 60-mm dishes were transfected as described above. After 48 h, cells were trypsinized, washed, resuspended in PBS/1% FBS (10^7 ^cells/ml) and filtered through a 40 *μ*m nylon mesh (BD Bioscience, San Jose, USA). Cells were purified from a pool of five 60-mm dishes using the FACS Vantage (Becton Dickinson, San Jose, CA) and the exclusion method at high speed (60 *μ*l/min). Cells were sorted using the settings previously described [17] and analyzed by analytical flow cytometry as described below. In general, 20,000 GFP+ cells were purified from 5 × 10^6 ^cells.

The percentage of GFP+ cells before and after purification by preparative cell sorting was determined by analytical flow cytometry using the FACS Vantage. All data acquisition and analyses were performed using the Cell Quest software (Becton Dickinson). To estimate the number of GFP+ cells, a FL1 histogram was generated (FL1, 530/30 nm short pass filter, detects green signal) and positive cells were defined as those cells in the region M1. The percentage of cells in M1 from the empty vector (mock)-transfected cells was subtracted from the percentage of plasmid-transfected cells in M1. Cell viability was determined using propidium iodide (PI) (Sigma) as reported [47]. PI fluorescence was detected with the FL2 emission channel (585/42 nm band pass filter). The percentage of dead cells was determined as the percentage of PI+ cells in a FL1 versus FL2 plot after subtracting the percentage of PI+ cells from the mock-transfected cells.

### DNA microarray analysis

The microarray analysis was performed as described in the Affymetrix expression analysis technical manual http://www.affymetrix.com. Total RNA (10 *μ*g) was extracted from three different cell populations: i) sorted TRH-GFP+ cells (GFP+); ii) TRH-GFP+ and GFP- mixed cells (GFP+/-) passed through the FACS but not sorted, and iii) non-transfected cells (NT). To obtain a sufficient amount of RNA for each cell population, the number of independent experiments pooled for the GFP+ sample was higher than for the other samples. Therefore, a pool of six independent experiments was used to prepare total RNA from the GFP+ and three independent experiments for GFP+/- or NT cells.

The microarray target was synthesized from total RNA. RNA was reverse-transcribed into double-stranded cDNA with a T7 promoter-containing primer using SuperScript II reverse transcriptase, RNase H, and DNA polymerase (Invitrogen). After precipitation with 5M NH_4_OAc and ethanol, the cDNA was used as a template in a biotin-labeled *in vitro *transcription reaction (Enzo BioArray, Affymetrix). Resulting target cRNA was collected on RNAeasy columns (QIAGEN, Valencia, CA) and then fragmented for hybridization on the microarrays.

The rat U34A microarray from Affymetrix was used. It contains probes for approximately 7699 well-annotated genes and around 1130 expressed sequence tags (ESTs) from *Rattus norvegicus*. Probes consist of 16 pairs of 25-mer oligonucleotides for each gene. One member of each pair contains a single base point mutation, and the signals of the pairs are compared to assess specificity of hybridization. Biotinylated target cRNA (15 *μ*g) was hybridized to the array and then processed using the Affymetrix GeneChip Fluidics Workstation 400. After binding with phycoerythrin-coupled avidin, microarrays were scanned on a Hewlett-Packard Gene Array Scanner (Hewlett-Packard Co., Palo Alto, CA). Data were deposited in the NCBI Gene Expression Omnibus repository http://www.ncbi.nlm.nih.gov/geo/ with the accession number GSE28441.

Results were analyzed using Affymetrix MAS 5.0 software. Individual microarrays were scaled to produce a mean signal intensity of 125. Iterative comparisons of different microarray datasets were done with MAS 5.0 comparison analysis as previously described with modifications [48]. To determine the expression difference between the GFP+ and GFP+/- cell populations, an additional approach was adopted. This involved grouping the two replicates of the control (i.e, GFP+/-) and the sorted sample (i.e, GFP+). Briefly, signal intensities of the two replicates of control and sorted datasets were averaged to represent the expression level of a transcript in the respective control and sorted populations. These averaged intensities were then used to calculate the fold enrichment in expression in sorted sample over control for each transcript. To identify transcripts that are enriched in one sample but underrepresented in the other, a threshold of more than 2 fold increase or decrease in expression was considered significant.

### RT-PCR

RNA was extracted as previously described [18]. Reverse transcription (RT) was performed with 1 μg of RNA using the M-MLV reverse transcriptase (Invitrogen) in the presence of oligo-dT_15 _primer. PCR was carried out in a total reaction volume of 50 μl. Primers (Additional file 1, Table S5) were designed using the PRIMER 3 software [49]. In general, PCRs were performed using 25 pmol of each of the specific forward and reverse primers, 1 μl of dNTP mix (20 mM each; Boehringer) and 1/5 of the RT reaction product.

Transcripts amplified by PCR included: *Krüppel-like factor 4*, *collagen type III alpha 1 *(Gene ID 84032), *up-regulated by 1,25 dihydroxyvitamin D-3 *(Gene ID 117514), *neurofilament heavy chain *[50], *green fluorescent protein *[51], *Trh*, *glyceraldehyde 3-phosphate dehydrogenase (g3pdh) *[18], *Tau *[52] and the *glial fibrillary acidic protein *[53]. Amplification was performed for 30 cycles except for *g3pdh *(24 cycles). PCR cycling conditions consisted of one cycle of melt temperature of 94°C for 1 min, a primer annealing step at 60°C or 64°C (*Klf4*) for 1 min, a polymerization step at 72°C for 1 min and a final extension at 72°C for 10 min. PCR products were electrophoresed in 2% agarose gel and bands stained with ethidium bromide.

## Authors' contributions

MG-C carried out primary cultures and transfection experiments, FACS, prepared the RNA for pooling and validation by RT-PCR, CP-M assisted with the data analysis and participated in writing the manuscript, SCJ performed the majority of the data analysis, SC-R assisted with the data analysis, RMG-R assisted with some data analysis and PJ-B participated in the design of the study, LP-M and J-LC conceived the study, participated in the design and coordination of the study and wrote the manuscript. All authors read and approved the final manuscript.
